# Effects of Medium Temperature and Industrial By-Products on the Key Hardened Properties of High Performance Concrete

**DOI:** 10.3390/ma8125464

**Published:** 2015-12-10

**Authors:** Md. Safiuddin, Sudharshan N. Raman, Muhammad Fauzi Mohd. Zain

**Affiliations:** 1 Angelo Del Zotto School of Construction Management, George Brown College, 146 Kendal Avenue, Toronto, ON M5T 2T9, Canada; 2 Department of Architecture, Faculty of Engineering and Built Environment, Universiti Kebangsaan Malaysia, 43600 UKM Bangi, Selangor Darul Ehsan, Malaysia; fauzizain@ukm.edu.my

**Keywords:** compressive strength, dynamic modulus of elasticity, high performance concrete, industrial by-products, initial surface absorption, medium temperature, moisture movement

## Abstract

The aim of the work reported in this article was to investigate the effects of medium temperature and industrial by-products on the key hardened properties of high performance concrete. Four concrete mixes were prepared based on a water-to-binder ratio of 0.35. Two industrial by-products, silica fume and Class F fly ash, were used separately and together with normal portland cement to produce three concrete mixes in addition to the control mix. The properties of both fresh and hardened concretes were examined in the laboratory. The freshly mixed concrete mixes were tested for slump, slump flow, and V-funnel flow. The hardened concretes were tested for compressive strength and dynamic modulus of elasticity after exposing to 20, 35 and 50 °C. In addition, the initial surface absorption and the rate of moisture movement into the concretes were determined at 20 °C. The performance of the concretes in the fresh state was excellent due to their superior deformability and good segregation resistance. In their hardened state, the highest levels of compressive strength and dynamic modulus of elasticity were produced by silica fume concrete. In addition, silica fume concrete showed the lowest level of initial surface absorption and the lowest rate of moisture movement into the interior of concrete. In comparison, the compressive strength, dynamic modulus of elasticity, initial surface absorption, and moisture movement rate of silica fume-fly ash concrete were close to those of silica fume concrete. Moreover, all concretes provided relatively low compressive strength and dynamic modulus of elasticity when they were exposed to 50 °C. However, the effect of increased temperature was less detrimental for silica fume and silica fume-fly ash concretes in comparison with the control concrete.

## 1. Research Significance

There are many ways of exposing concrete structural members to a medium temperature in the range of 20–50 °C. Some of the most common types of concrete exposed to such a medium temperature are those used in power plants, chemical plants, and nuclear reactors. Also in hot weather, where the temperature is sometimes over 35 °C, direct sunrays can increase the temperature of concrete members up to 50 °C. In these cases, the properties of high performance concrete may undergo distinct changes. Generally, at a temperature of 50 °C, the movement of moisture from the concrete surface could affect the hardened properties of concrete, resulting in increased drying shrinkage cracking. Also, microcracks might appear due to thermal stress and thus would affect the hardened properties of concrete. Therefore, this study investigated the compressive strength and dynamic modulus of elasticity of different high performance concretes exposed to a medium temperature range of 20–50 °C. Furthermore, the initial surface absorption and the rate of moisture movement into concrete are linked with its pore structure or microstructure and therefore may act as the key indicators for the durability performance of concrete in service conditions. The incorporation of certain industrial by-products could enhance the durability performance of concrete by reducing water absorption or moisture movement into concrete. Hence, this study also examined the initial surface absorption and the rate of moisture movement into different high performance concretes incorporating two industrial by-products, namely silica fume and fly ash.

## 2. Introduction

High performance concrete (HPC) has opened manifold opportunities for the construction of concrete structures. According to Forster [1], “High performance concrete is a concrete made with appropriate materials combined according to a selected mix design and properly mixed, transported, placed, consolidated and cured so that the resulting concrete gives excellent performance in the structure in which it will be placed, in the environment to which it will be exposed and with the loads to which it will be subjected for its design life”. Hence, HPC should be workable, pumpable, and easily compactable within the confines of any formwork or reinforcement. After a proper curing regime, it must achieve the desired level of strength to carry the expected loads during its design life. Also, it must withstand different detrimental factors when exposed to the environment.

HPC can be made with the same basic materials used for ordinary concrete; the major differences are the mix proportions and water-to-binder (W/B) ratio. The W/B ratio of HPC is much lower than that of ordinary concrete. While ordinary concrete has a W/B ratio between 0.50 and 0.75, it ranges from 0.20 to 0.35 for HPC [2]. This reduction in W/B ratio is not only obtained by increasing the amount of cement, but also by simultaneously decreasing the amount of mixing water in the presence of superplasticizer (SP). In addition, the use of a suitable supplementary cementitious material (SCM) such as silica fume or fly ash improves the strength and durability of HPC. In particular, the use of silica fume is essential for obtaining very high strength HPC [3]. Most SCMs are obtained as industrial by-products. For example, silica fume is obtained as a by-product of silicon and ferrosilicon alloy production whereas fly ash is obtained as one of the residues generated by coal combustion in power plants. The use of SCMs in concrete provides both economic and environmental benefits as they decrease the demand for cement and eliminate the disposal problems caused by industrial wastes. The successful use of SCMs obtained as industrial by-products would reduce the environmental load to the landfill sites as well as the greenhouse gas emissions from cement factories.

HPC has been used to construct many novel concrete structures in the world. In 1988, 120 MPa HPC was used in Seattle, USA to construct the columns for a 52-storey building at 2 Union Square [4]. In the same year, 100 MPa HPC went into the columns of one of the world’s tallest concrete building [5]. In 1984, an experimental column was poured in the La Laurentienne building, Montreal with 90 MPa HPC [6]. Toronto’s Scotia Plaza, completed in 1987, had HPC with an average compressive strength of 93 MPa [7]. The Hibernia Gravity Base Structure, constructed in Newfoundland, Canada, incorporated HPC for strength and durability [5]. In Europe, HPC was stated to be developed for the construction of large offshore platforms and bridges, but very rarely for the construction of high-rise buildings [2]. HPC was used to build huge offshore structures in Norway [8]. Later HPC was used to construct highway pavement in Norway due to its superior resistance to abrasion [9,10]. In France, many researches were conducted in the Laboratorie Central de Recherche [11] to master the characteristics and behaviour of HPC. Moreover, the Institute of Reinforced and Prestressed Concrete (IBAP) at the Ecole Polytechnique Federale in Lausanne undertook several research activities concerning the serviceability of concrete structures. All of those efforts produced advances in the scientific and technological fields of HPC [12]. In Asia, Japan made a significant contribution to the development of HPC. The researchers from the concrete laboratory of the University of Tokyo in Japan developed super-workable HPC [13]. The Southeast Asian countries also used high strength HPC. In the Kuala Lumpur City Centre (KLCC) project, Malaysia, 80 MPa high strength HPC was used. In the Bioyette Tower of Bangkok, Thailand, 80 MPa high strength HPC was also used to reduce the cost and ease the construction [14].

One of the most essential practices that must be given high priority for stringent quality control in the production of HPC is the curing of concrete at a proper temperature. Many researchers have investigated the effects of curing technique, temperature, and environmental and climatic impacts on the mechanical and durability characteristics of various concretes including HPC [15,16,17,18,19,20,21,22,23]. Ait-Aider *et al.* [15] reported based on their findings that under hot climates, increasing the water amount in the concrete mix to a certain limit can contribute positively by maintaining the required workability of the mix and compensating the water amount that was lost by evaporation. This additional quantity of water did not show any marked detrimental effect on the strength of the hardened concrete [15]. Meanwhile, Al-Gahtani *et al.* [16] found that, for blended cement concretes produced with Type I cement (normal portland cement), silica fume and fly ash, the concrete specimens cured by wet burlap covering provided more promising results in terms of strength development, in comparison with the specimens cured with water-based or acrylic-based curing compounds. However, the specimens cured with acrylic-based curing compounds indicated greater efficiency in reducing the plastic and drying shrinkages compared with the concrete specimens cured with wet burlap or a water-based curing compound. Ibrahim *et al.* [18] researched the contribution of different curing techniques to the strength and durability characteristics of normal portland cement and silica fume concretes. They found that the strength and durability properties of the concrete specimens cured with selected curing compounds were superior compared with the specimens cured using wet burlap. The performance of different concretes was analysed based on their compressive strength, water absorption, and chloride permeability. While there was no significant distinction in terms of strength observed, the specimens cured with a bitumen-based curing compound exhibited the best durability characteristics with respect to water absorption and chloride permeability. Nassif and Petrou [19] found that the curing of concrete at very low, near-freezing temperatures may result in 20% and 25% loss in the 28-day stiffness and strength of the concrete, respectively, and the replacement of 20% portland cement with fly ash cannot compensate for the detrimental effects of cold weather concreting. This was established after experimental tests on the concrete slab specimens under various curing regimes between 20 °C and −5 °C. Furthermore, Nie *et al.* [20] researched the feasibility of using local mineral admixtures as supplementary cementitious material in concrete subjected to severe sulphate environmental conditions in northwest China. The chemical, mechanical, and durability characteristics of concrete were analysed in their study. They reported that the incorporation of mineral admixtures such as Class F fly ash and slag improves the resistance of concrete to the harsh environment [20].

The temperature in concrete and surroundings are expected to influence the performance of different concretes including HPC [15,18,21,22]. The increased temperature of both fresh concrete mix and the surrounding air during the execution of concrete work influences the properties of hardened concrete. The increased temperature of curing affects the compressive strength, elasticity, and durability properties of HPC. This is because the increase in temperature affects the hydration of cement in concrete [15,18,19,22]. The hydration of cement decreases significantly when the ambient relative humidity during curing of concrete is below 80% [24]. Therefore, the early drying of concrete at higher temperature may stop the cement hydration before the capillaries are blocked by hydration products. The covercrete is more sensitive to drying since it is more prone to losing water. The formation of a network of capillaries in covercrete may provide easy passage for the intrusion of aggressive species that cause deterioration of the concrete structures [25]. Early drying can also lead to more shrinkage cracking and this would aggravate the deterioration process of concrete. Hence, a favorable temperature must be maintained while curing the concrete, especially during the early period for preventing premature drying and interrupted cement hydration. Moreover, a disconnected pore structure or a compact microstructure with reduced porosity is required to enhance the durability performance of concrete. This can be achieved by curing the concrete at a proper temperature.

The present study investigated the effects of three different medium temperatures of 20, 35 and 50 °C as well as two industrial by-products, silica fume and fly ash, on the key hardened properties such as compressive strength and dynamic modulus of elasticity of HPC. The hardened concrete specimens were tested for the aforementioned hardened properties after curing in water at 20 °C for selected periods (3, 7 and 14 days) and then exposing them to a medium temperature of 35 or 50 °C until the day of testing. A number of concrete specimens water cured at 20 °C were also tested to observe the effect of silica fume and fly ash on the initial surface absorption (ISA) and moisture movement into concrete.

## 3. Experimental Investigation

### 3.1. Constituent Materials

Coarse aggregate (locally available crushed granite stone), fine aggregate (locally available mining sand), normal portland cement (locally available ASTM Type I cement), tap water, silica fume (imported microsilica), fly ash (locally available ash), and SP were used to produce the concrete mixes. The maximum size of coarse aggregate was 19 mm and that of fine aggregate was 4.75 mm. The bulk specific gravity of coarse aggregate and fine aggregate was 2.62 and 2.60, respectively. The absorption of fine aggregate and coarse aggregate was 1.20% and 0.90%, respectively. The unit weight of fine aggregate was 1720 kg/m^3^ and that of coarse aggregate was 1550 kg/m^3^. Both coarse aggregate and fine aggregate fulfilled the grading requirements of ASTM C33/C33M-13 [26]. The fineness modulus of fine aggregate and coarse aggregate was 3.01 and 6.64, respectively.

The cement required for this study was taken from the same batch of production to avoid additional variables. The cement conformed to the requirements of normal portland cement stated in ASTM C150/C150M-15 [27]. The specific gravity of cement was 3.15. Silica fume that had been used in the study was Elkem Microsilica, Grade 920-D. The specific gravity and the average particle size was 2.20 and 0.1 μm, respectively. Fly ash used in this study was collected from a local power plant located in the state of Selangor, Malaysia. It was Class F fly ash in accordance with the specifications of ASTM C618-12a [28]. The specific gravity of fly ash was 2.26. A Type F sulfonated naphthalene formaldehyde condensate based SP conforming to ASTM C494/C494M-13 [29] was used in this study. It was available in dark brown aqueous solution. It contained no chloride. The specific gravity was 1.21 and the solid content was 40%. Normal tap water (pH = 6.9) was used for mixing all concretes. Tap water was also used to cure the concretes. The mix water used in producing different concretes complied with the requirements set forth in BS EN 1008: 2002 [30].

### 3.2. Concrete Mix Proportions

Four different concrete mixes using a W/B ratio of 0.35 were produced. The Sherbrooke mix design method [31] was followed and a number of trial mixes were conducted to decide the proportions of constituent materials for different concretes. The details of the mix proportions for different concretes are given in Table 1. The concrete mixes were designated according to their mix compositions. The designations NPCC, SFC, FAC and SFFAC refer to normal portland cement concrete, silica fume concrete, fly ash concrete and silica fume-fly ash concrete, respectively.

The concrete mixes were proportioned to have a minimum slump of 19 cm and a minimum slump flow of 50 cm. The W/B ratio of 0.35 was used to fulfill the minimum strength requirement (50 MPa) of HPC [32]. The mix water content was 185.5 kg for all concrete mixes. This water content was above the minimum water content as recommended for HPC [31]. The binder (cement plus supplementary cementitious material) content was 530 kg/m^3^ for all concrete mixes. The binder content of HPC generally ranges from 392 kg/m^3^ to 557 kg/m^3^ [33]. The coarse aggregate content of different concrete mixes was in the range of 1002–1013 kg/m^3^. For HPC, the coarse aggregate content normally ranges from 950 kg/m^3^ to 1150 kg/m^3^ for different particle shapes [31]. The fine aggregate content in all concrete mixes was about 40% of the total aggregates (fine aggregate plus coarse aggregate).

**Table 1:** Details of concrete mix proportions.

Concrete Type	Coarse Aggregate (kg/m^3^_)_	Fine Aggregate (kg/m^3^)	Binder (B, kg/m^3^)			Water Content (kg/m^3^)	SP (%B)
			Cement	Silica Fume	Fly Ash		
NPCC	1013	675	530	–	–	185.5	1.75
SFC	1002	667	477	53	–	185.5	2.25
FAC	1003	668	477	–	53	185.5	1.75
SFFAC	1002	668	477	26.5	26.5	185.5	2.15

Note: The W/B ratio of all concrete mixes was 0.35.

Silica fume and fly ash were used to produce three HPC mixes (namely SFC, FAC and SFFAC). In SFC mix, silica fume was used by replacing 10% of cement on weight basis. Theoretically, the optimum silica fume content for obtaining high strength is around 20% to 25% of cement by weight. However, considering the cost of silica fume and other admixtures, the practical and economic optimum content is closer to 10% by weight of cement [34]. Therefore, 10% silica fume by weight of cement was used in SFC mix. In FAC mix, fly ash was used by replacing 10% of cement by weight. Besides, in SFFAC mix, 5% cement was replaced by silica fume and another 5% cement was replaced by fly ash. The SP dosages used for different concrete mixes were in the range of 1.75% to 2.25% by weight of binder (cement plus silica fume and/or fly ash). These SP dosages were determined based on the workability of trial concrete mixes.

### 3.3. Preparation of Fresh Concrete

The constituent materials were mixed in a rotating pan type mixer (capacity 0.05 m^3^) conforming to BS 1881: Part 125: 2013 [35]. The batch volume of concrete was calculated based on the number of test specimens and the amount of concrete required for testing fresh properties (slump, slump flow and V-funnel flow). The quantity of concrete in each batch was at least 15% more than the required volume. In all concrete batches, the fine and coarse aggregates were measured on an air-dry weight basis. Also, cement was taken based on weight. However, the water and SP were measured based on volume. The water corrections were done considering the absorption of fine and coarse aggregates as well as the water contributed by liquid SP.

### 3.4. Testing of Key Fresh Properties of Concrete

Immediately after the completion of mixing, the samples of freshly mixed concrete were taken for testing slump, slump flow, and V-funnel flow. Slump and slump flow were measured according to ASTM C143/C143M-12 [36]. A sample of freshly mixed concrete was placed and compacted by rodding in a mould shaped as the frustum of a cone (slump cone). After completing the filling operation, the slump cone was raised and the concrete was allowed to subside. The distance between the original and displaced positions of the centre of the top concrete surface was measured and reported as the slump of the concrete. The average diameter of the deformed concrete sample was also measured from the same test and reported as the slump flow of the concrete.

**Figure 1:**
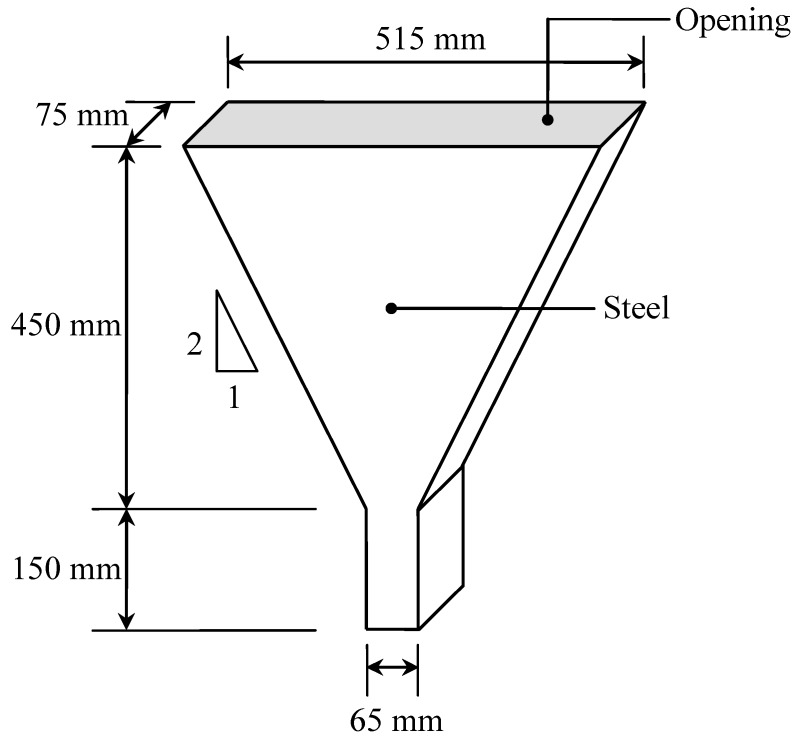
V-funnel apparatus.

The flowability of the concrete mixes with respect to flow time was also determined in the present study. A V-funnel apparatus (refer to Figure 1) was used to determine the flowability of concrete in accordance with Japanese Industrial Standard JIS R 5201: 1993 [37]. The capacity of the V-funnel was 10.5 L. The constricted end had an opening of 75 mm × 65 mm. A sample of freshly mixed concrete (10.5 L in volume) was placed in the V-funnel apparatus without any interruption keeping the lower opening closed. Then the lower opening was made free and the concrete was allowed to flow from the V-funnel. The flow time of the concrete was measured by a stopwatch. The noted flow time (V-funnel flow) was used as a measure for the flowability of fresh concrete.

### 3.5. Fabrication of Hardened Test Specimens

Cylinder specimens of Ø100 mm × 200 mm were fabricated for use in tests to obtain compressive strength, dynamic modulus of elasticity, and moisture movement rate. In addition, 150 mm cube specimens were fabricated for the ISA test. Both the cylinder and cube specimens were moulded in reusable cast-iron moulds. The moulding of test specimens from fresh concrete was carried out following BS EN 12390-2: 2009 [38] with the exception of using two layers of filling instead of three. The reason is that the fresh concrete was highly workable. A vibrating table was used for a short time for compaction during moulding. The capping operation was carried out for the freshly moulded cylindrical specimens to get plane and parallel end surfaces. Immediately after moulding and finishing, all test specimens were covered with a plastic sheet to prevent evaporation of water from unhydrated specimens and kept in a comparatively cool place under shade. The test specimens were removed from the moulds at the age of 24 ± 2 h.

### 3.6. Curing and Conditioning of Hardened Test Specimens

The Ø100 mm × 200 mm cylinder specimens and the 150 mm cube specimens were cured in water. After demoulding, the test specimens were marked, weighed and stored inside the water tank situated in an air-conditioned room maintaining the temperature at 20 °C. After 3, 7 and 14 days, the cylinder specimens in required numbers were transferred to the ovens operated at the temperatures of 35 and 50 °C. It should be mentioned that a set of Ø100 mm × 200 mm cylinder specimens was continued to cure in water tank at 20 °C until the day of testing so that the effect of exposure to 35 and 50 °C medium temperatures could be clearly observed for the compressive strength and dynamic modulus of elasticity of the concretes. Another set of Ø100 mm × 200 mm cylinder specimens was kept in water at 20 °C to determine the rate of moisture movement into the concretes. Also, a set of 150 mm cube specimens was continuously water-cured at 20 °C for use in testing the ISA of the concretes.

### 3.7. Testing of Key Hardened Properties of Concrete

The compressive strength of the concretes was examined at the ages of 3, 7, 14, 28, and 91 days according to ASTM C39/C39M-14a [39]. The dynamic modulus of elasticity of the concretes was determined at the ages of 3, 7, 14, 28, and 91 days according to BS 1881: Part 209: 1990 [40] by using an Erudite MK II Resonant Frequency Test System. The dynamic modulus of elasticity was obtained based on the fundamental longitudinal resonant frequency recorded from the aforementioned test system. The ISA of the concretes was determined at the age of 28 days in accordance with BS 1881: Part 5: 1970 [41]. The rate of moisture movement into the concretes was determined at the ages of 3, 7, 14, 28, and 91 days based on the weight gain during continuous water curing at 20 °C.

## 4. Results and Discussion

### 4.1. Fresh Properties of Concrete

The properties of freshly mixed concretes were determined with respect to slump, slump flow, and V-funnel flow. These are given in Table 2. All concretes possessed superior deformability and good segregation resistance and therefore provided the required slump, slump flow, and V-funnel flow. Adequate SP dosage contributed to increase the deformability of concrete without any visible form of segregation such as bleeding, paste separation, mortar halo, and aggregate hump. The SP dosage varied depending on the type of SCM used in concrete. It was observed that SFC required the highest SP dosage to achieve the required flowability of HPC. This is because the water demand of SFC for given flowability increased in the presence of silica fume. Silica fume with a lower particle size and a higher specific surface area necessitates a relatively high amount of water to obtain the required flowability [42]. Since the water content was fixed at 185.5 kg/m^3^, a greater SP dosage was required to compensate for the higher water demand.

**Table 2:** Key fresh properties of different concrete mixes.

Concrete Type	Water Content (kg/m^3^)	SP (%B)	Slump (cm)	Slump Flow (cm)	V-Funnel Flow (s)
NPCC	185.5	1.75	25.0	58.0	55.3
SFC	185.5	2.25	25.0	58.0	58.3
FAC	185.5	1.75	23.5	53.0	87.5
SFFAC	185.5	2.15	24.0	55.0	65.6

### 4.2. Hardened Properties

The compressive strength, dynamic modulus of elasticity, and moisture movement rate of all concretes were determined at 3, 7, 14, 28 and 91 days after water curing at the temperature of 20 °C. The compressive strength and dynamic modulus of elasticity of each type of concrete were also determined at the ages of 28 and 91 days after 3, 7 and 14 days of water curing followed by exposure to two medium temperatures, 35 and 50 °C. The ISA of all concretes were determined at the age of 28 days after uninterrupted water curing at 20 °C.

#### 4.2.1. Initial Surface Absorption

The results of ISA test for different concretes are given in Table 3. These results were found by testing three companion 150 mm cube specimens for each type of concrete. The test point time periods were 10, 30, 60 and 120 min from the start of the test. The difference between the mean and respective ISA values was 2% to 4%.

Amongst the four types of concrete, the ISA of FAC was slightly lower than that of NPCC. However, SFC exhibited the best resistance to water penetration by giving the least ISA-value. This finding is consistent with the finding reported from an earlier research [25]. The results also showed that SFFAC provided lower ISA than FAC. Hence, the overall ISA results indicate that the high pozzolanic reactivity and micro-filling effect of silica fume modified the open channels in the bulk paste and transition zone of SFC, making it much denser and leading to a fine and discontinuous pore structure. The significant decrease in ISA in the presence of silica fume is due to two phenomena; the first one is physical and the second one is chemical [43]. The physical phenomenon acts immediately at early ages when the chemical phenomenon is still latent. According to Detwiler and Mehta [43], the physical phenomenon is attributable to the fineness of silica fume, its large specific surfaces, and to the fact that its particles fill in the existing spaces between various granules of cement and those between cement paste and aggregate, which are rich in exuded water and calcium hydroxide (liberated from cement hydration). Later, the chemical reaction between silica fume and calcium hydroxide comes into action producing additional calcium silicate hydrate (CSH), which occupies the larger spaces between two reagents and thus reduces the porosity of concrete.

**Table 3:** Initial surface absorption of different concretes.

Concrete Type	ISA Rate (×10^−2^ mL/m^2^/s) after			
	10 min	30 min	60 min	120 min
NPCC	30	21.9	16.4	12.2
SFC	25	13.2	8.7	4.3
FAC	29	20.6	13.8	8.6
SFFAC	24.3	15.5	11.0	6.9

#### 4.2.2. Moisture Movement

The test results for the rate of moisture movement into different types of concrete are shown in Table 4. These results were found by testing three companion Ø100 mm × 200 mm cylinder specimens for each type of concrete. The difference between the mean and individual moisture movement rates was 5% to 10%.

**Table 4:** Moisture movement into different concretes.

Concrete Type	Rate of Moisture Movement (×10^−3^ kg/day) after				
	3 Days	7 Days	14 Days	28 Days	91 Days
NPCC	7.8	5.4	3.7	2.6	0.5
SFC	3.0	2.0	1.2	0.3	0.2
FAC	6.7	3.8	2.1	1.3	0.4
SFFAC	4.5	2.4	1.9	1.1	0.3

Test results showed that the incorporation of SCM into concrete influences the moisture movement rate of concrete. In this study, all SCM concretes have shown less moisture movement than NPCC. Among SCM concretes, the moisture movement rate of SFFAC was lower than that of FAC. However, the least moisture movement occurred in the case of SFC. Hence, it indicates that the porosity was significantly reduced in SFC as compared with other concretes due to pore refinement made by silica fume. Due to the micro-filling and pozzolanic effects of silica fume, as discussed in Section 4.2.1, the process of pore filling occurs in SFC. Thus, SFC possesses a fine and discontinuous pore structure, resulting in the lowest rate of moisture movement.

#### 4.2.3. Compressive Strength

**Figure 2:**
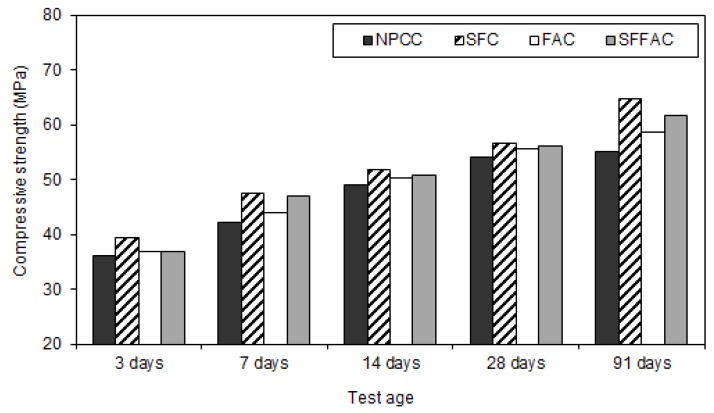
The compressive strength of different types of concrete at various ages after water curing at a medium temperature of 20 °C.

**Figure 3:**
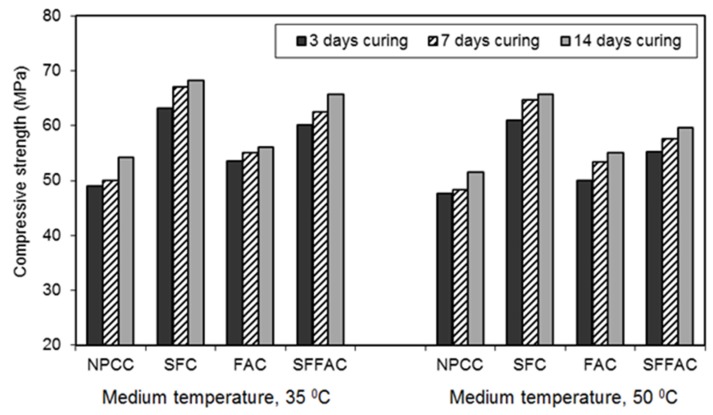
The 28-day compressive strength of different types of concrete transferred after 3, 7 and 14 days of water curing at 20 °C followed by exposure to two medium temperatures of 35 and 50 °C.

**Figure 4:**
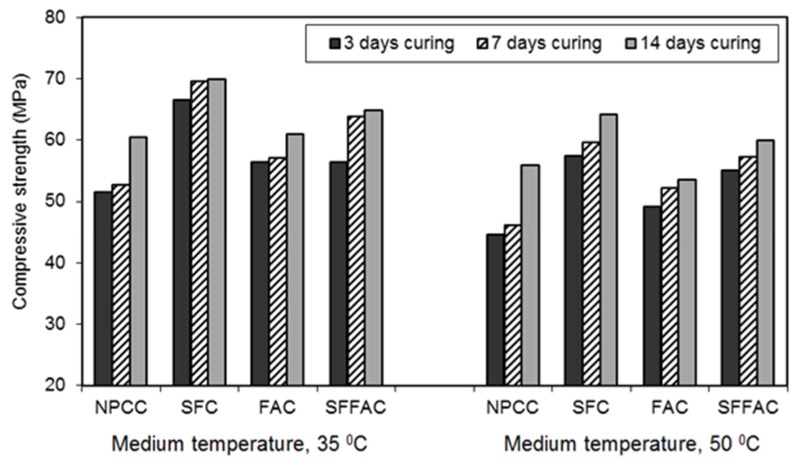
The 91-day compressive strength of different types of concrete transferred after 3, 7 and 14 days of water curing at 20 °C followed by exposure to two medium temperatures of 35 and 50 °C.

The results of the compressive strength test are presented in Figure 2, Figure 3 and Figure 4. These results were obtained by testing triplicate Ø100 mm × 200 mm cylinder specimens for each type of concrete. The difference between the average compressive strength and individual cylinder strength was 2.5% to 3.5%. The compressive strength practically increased up to 91 days for all types of concrete (refer to Figure 2). In all concretes, the concrete specimens that were transferred after 7 and 14 days of water curing showed better gain in compressive strength at 28 and 91 days (refer to Figure 3 and Figure 4). In general, 14 days of water curing produced the highest compressive strength. This is because more moisture was available for additional days to continue cement hydration, thus resulting in more hydration products that contributed to increase the compressive strength of concrete. The greater amount of hydration products decreased the capillaries in concrete by pore blocking and therefore increased the compressive strength. Moreover, it was observed that 7 days of water curing produced the compressive strength close to that provided by 14 days of water curing. This suggests that 14 days of water curing is the best for concrete made with normal portland cement but the minimum curing age should be at least 7 days.

The compressive strength of each type of concrete exposed to the temperature of 50 °C after 3, 7 and 14 days of water curing was lower than that obtained at the exposure temperature of 35 °C. Hence, the compressive strength of concrete decreased with the increase in temperature. This is because an elevated curing temperature causes a lower degree of cement hydration due to a loss of water from concrete. A certain amount of the pore water dries out from concrete during drying. Hence, some cement particles may remain unhydrated, resulting in a lower strength. Similar results were found in an earlier study [25]. The hydration of cement in concrete significantly decreases when the ambient relative humidity drops below 80% [24]. This is likely to occur when the concrete specimens are exposed to dry air with a temperature of 50 °C.

All types of concrete containing SCM showed higher strength achievement than NPCC (control concrete). However, the strength achievement was the highest in the case of SFC. Test results show that after 91 days and at the temperature of 35 °C, SFC produced a compressive strength of 70 MPa. Silica fume is a highly reactive SCM. It readily reacts with water to produce additional hydration products (pozzolanic reaction products). The open channels in SFC are blocked by the high pozzolanic reaction products of silica fume. Besides, silica fume has the micro-filling ability because of its extremely small particle size, as discussed earlier in Section 4.2.1 and Section 4.2.2. Hence, it fills in the microvoids between cement particles. Moreover, an enhanced bonding strength develops between cement paste and aggregate because of the accelerated pozzolanic and micro-filling actions of silica fume. These are the probable reasons why SFC exhibited the highest gain in compressive strength as compared with other concretes.

All SCM concretes were less sensitive to the increased curing temperatures. At the medium temperature of 50 °C, SCM concretes produced higher compressive strength than NPCC. This observation is clearly perceptible in Figure 3 and Figure 4. This is due to the reason that the use of SCM results in porosity reduction, pore refinement, and decreased water movement, as understood based on the results of ISA and moisture movement tests. Besides, amongst SCM concretes, SFFAC exhibited higher compressive strength than FAC but lower compressive strength than SFC. FAC produced the lowest compressive strength amongst three SCM concretes. This observation indicates that fly ash alone is not very efficient to increase the compressive strength of concrete. However, the combined use of silica fume and fly ash can accelerate the increase in compressive strength. This is due to the fact that silica fume has better pozzolanic activity and micro-filling ability than fly ash, as discussed in Section 4.2.1 and Section 4.2.2.

#### 4.2.4. Dynamic Modulus of Elasticity

**Figure 5:**
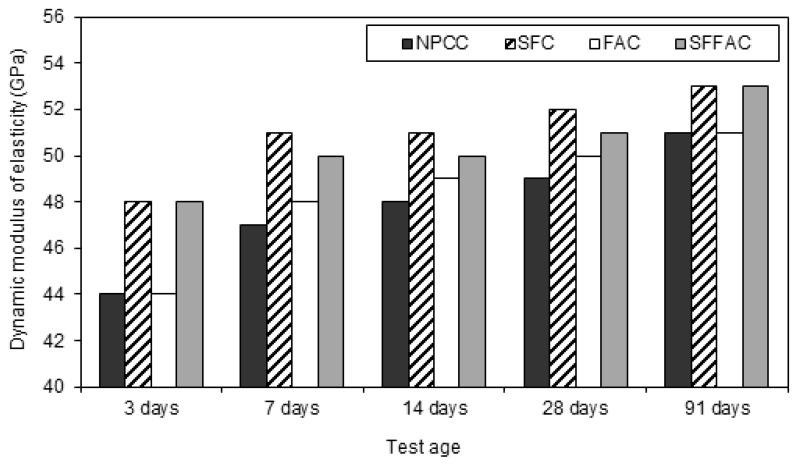
The dynamic modulus of elasticity of different types of concrete at various ages after water curing at a medium temperature of 20 °C.

**Figure 6:**
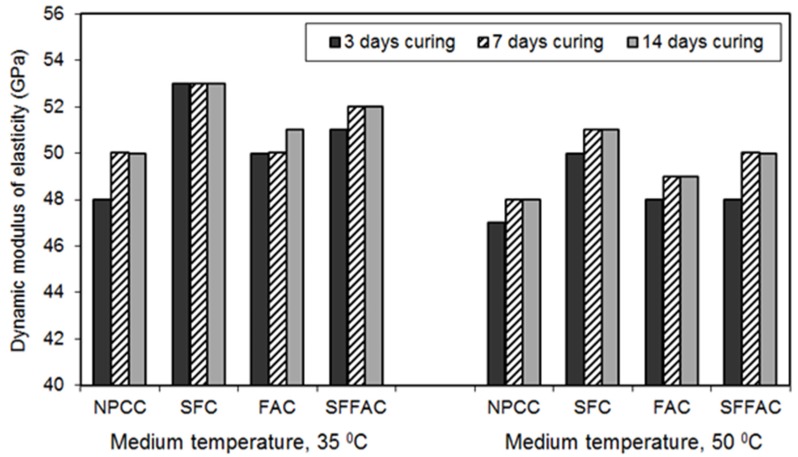
The 28-day dynamic modulus of elasticity of different types of concrete transferred after 3, 7 and 14 days of water curing at 20 °C followed by exposure to two medium temperatures of 35 °C and 50 °C.

**Figure 7:**
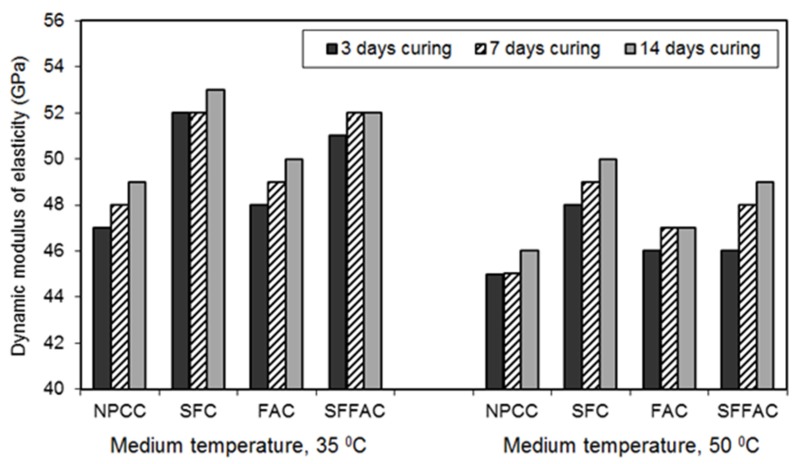
The 91-day dynamic modulus of elasticity (GPa) of different types of concrete transferred after 3, 7 and 14 days of water curing at 20 °C followed by exposure to two medium temperatures of 35 °C and 50 °C.

The results of the dynamic modulus of elasticity test for different types of concrete are presented in Figure 5, Figure 6 and Figure 7. These results were obtained from testing triplicate Ø100 mm × 200 mm concrete cylinders for each type of concrete. The difference between the average and individual dynamic moduli of elasticity was 2% to 3%.

Test results show that the dynamic modulus of elasticity increased continuously up to 91 days when curing was carried out at 20 °C. It was also observed that the continuous water curing up to 91 days exhibited the highest dynamic modulus of elasticity compared with 7 days and 14 days of water curing (refer to Figure 5). Hence, it was understood that the dynamic modulus of elasticity significantly depends on the moisture content of concrete. Moreover, concerning transfer ages after water curing, it was found that 7 and 14 days of water curing provided higher dynamic modulus of elasticity than 3 days of water curing. The longer water curing produced more hydration products from cement hydration and thus made the microstructure of concrete denser due to reduction in porosity and pore refinement, as explained earlier in Section 4.2.1 and Section 4.2.2. This suggests that HPC should be cured at least for 7 days or preferably for 14 days by spraying with water or by covering with wet burlap.

All SCM concretes produced a higher dynamic modulus of elasticity than NPCC at all ages and temperatures (refer to Figure 5, Figure 6 and Figure 7). The increase in dynamic modulus of elasticity in the SCM concretes is related to the efficiency of SCM. The efficiency of SCM results from the role it plays as a micro-filler and porosity reducer [18,44]. Besides, the efficiency depends upon water availability during hydration, curing period, and exposure to temperature. The results of ISA and moisture movement tests indicate that SFC had the lowest porosity and most refined pore structure (refer to Section 4.2.1 and Section 4.2.2). This means that silica fume was most efficient in reducing the loss of moisture from concrete, and therefore SFC provided the highest level of dynamic modulus of elasticity. Indeed, this is due to the role played by silica fume as an effective micro-filler and porosity reducer at the early ages of concrete. Moreover, the presence of silica fume in SFFAC acted more crucially than fly ash to increase the dynamic modulus of elasticity. This also reveals the essential role of silica fume in producing HPC.

The exposure temperature affected the dynamic modulus of concrete. At 35 °C and 50 °C, the values of dynamic modulus of elasticity decreased after 28 days for all concrete types (compare Figure 6 and Figure 7). The dynamic modulus of elasticity also dropped continuously with the length of exposure to medium temperatures. Furthermore, the dynamic modulus of elasticity of all concrete types decreased more for the higher exposure temperature (refer to Figure 6 and Figure 7). Similar results were observed in past research [45,46]. The reduction in the dynamic modulus of concrete in such cases is linked with the moisture movement from the specimens. The concrete dries up more with the increased temperature and length of exposure as more moisture moves out of the specimens. The moisture loss interrupts cement hydration, resulting in a lower amount of hydration products. Hence, the pore-structure of concrete may not be as refined as that obtained through continued water curing at 20 °C. It was observed in an earlier research that the presence of larger and more pores in paste decreases the elastic modulus of concrete [47].

### 4.3. Effect of Medium Temperature on Hardened Properties

The effect of medium temperature depends on curing periods and moisture content. In this study, the effects of three medium temperatures, 20, 35 and 50 °C were studied. After 3, 7, and 14 days of water curing at 20 °C, the concrete specimens were transferred to two ovens and dried at the medium temperatures of 35 and 50 °C. The dried specimens were tested for compressive strength and dynamic modulus of elasticity at the ages of 28 and 91 days. Test results show that the temperature environment at 35 °C produces higher compressive strength and dynamic modulus than the temperature environment at 50 °C. This implies that the numerical values of the two above-mentioned hardened properties decrease with the increase in temperature. This is because the exposure to a higher medium temperature increases the moisture movement (moisture loss), which affects the microstructure of concrete due to interrupted cement hydration as discussed in Section 4.2.1 and Section 4.2.2. Moreover, it was observed that 7 and 14 days of initial water curing produced better results at the ages of 28 and 91 when the concrete specimens were dried at 35 °C (after transfer ages). Hence, the most effective way to achieve the expected strength and elasticity of concrete is to maintain its surrounding temperature at ≤35 °C at least for 7 days or preferably for 14 days at the early ages of hydration.

### 4.4. Effect of Supplementary Cementitious Materials on Hardened Properties

All SCM concretes exhibited higher compressive strength and dynamic modulus of elasticity as well as lower ISA and moisture movement rate than NPCC. However, amongst SCM concretes, silica fume concrete produced excellent performance. The incorporation of SCMs increases the number of fine particles in the system [48]. The presence of these fine particles contributes to the increased density of cement matrix in concrete. Also, SCMs create a discontinuous pore structure and clog pore channels with additional hydration products. The consumption of water-soluble calcium hydroxide (produced as a by-product during the hydration of cement) in the pozzolanic reaction with SCMs creates additional calcium silicate gel, which is much denser than the gel obtained from cement hydration [49]. As a result, SCM concretes gain better strength and elasticity as compared with NPCC.

SCMs influence the behavior and characteristics of the transition zone in concrete. When a concrete is subjected to stresses including thermal stress, the cracks begin to develop in the transition zone. If large pores and microcracks are present in the transition zone, the aggregate simply plays no role in determining the strength of concrete. Therefore, by using SCMs especially silica fume, the weakest link in the transition zone can be controlled and thus the strength and other properties of concrete can be improved. Silica fume produces a denser transition zone and thereby enhances the physical role of the granular skeleton of concrete [50]. In this study, HPCs were produced by using SCMs at a lower W/B ratio of 0.35 and with the aid of an SP. The incorporation of SCMs such as silica fume significantly improved the hardened properties of concrete. SFC produced the highest compressive strength and dynamic modulus of elasticity plus the lowest ISA and moisture movement rate at all curing conditions as compared with NPCC.

## 5. Conclusions

The following conclusions were drawn from the results of this study regarding the effects of medium temperature and industrial by-products on the hardened properties of HPC:

1. The concrete mixes provided a slump, slump flow, and V-funnel flow as specified for HPC. The addition of silica fume required a higher dosage of SP than any other type of concrete to get the required flowability.
2. The period of water curing was a critical factor to reduce the detrimental effect of concrete drying at medium temperature. Fourteen (14) days of water curing before exposure to the medium temperatures of 35 and 50 °C greatly improved the hardened properties of concrete. This is because more moisture was available for additional days to continue cement hydration, thus resulting in more hydration products that further improved the hardened properties of concrete.
3. SCM concretes produced lower ISA and moisture movement rate than NPCC. Especially, SFC provided the lowest levels of ISA and moisture movement rate. These results were attributable to the physical (microfilling) and chemical (pozzolanic reaction) effects of silica fume that contribute to produce a refined pore structure and decrease the porosity of concrete.
4. SCM concretes produced greater compressive strength and dynamic modulus of elasticity than NPCC. In particular, silica fume concrete provided the highest levels of compressive strength and dynamic modulus of elasticity when exposed to medium temperatures. This indicates that the accelerating pozzolanic reaction of SCM (particularly silica fume) at medium temperatures increases the strength and improves the quality of concrete.
5. The compressive strength and dynamic modulus of elasticity of the concretes dried at 35 °C were higher than those of the concretes dried at 50 °C. This indicates that the compressive strength and dynamic modulus of elasticity of concrete decreased as the temperature increased. However, the effect of temperature rise was less detrimental in SCM concretes than NPCC.
6. The concrete element exposed to a higher medium temperature should be well-cured to ensure high performance of concrete. The evaporation of water from concrete at an early age must be prevented to avoid the decrease in strength, modulus of elasticity, and other hardened properties of concrete. The suitable medium temperature environment associated with an adequate period of water curing (at least 7 days; preferably 14 days) plays an important role in the hydration process to produce the concrete with desired hardened properties.
